# Efficacy and Safety of Sanfu Herbal Patch at Acupoints for Persistent Allergic Rhinitis: Study Protocol for a Randomized Controlled Trial

**DOI:** 10.1155/2015/214846

**Published:** 2015-08-02

**Authors:** Xiankun Chen, Chuanjian Lu, Cecilia Stålsby-Lundborg, Yunying Li, Xiaoyan Li, Jian Sun, Wenwei Ouyang, Geng Li, Guobin Su, Liming Lu, Wenbin Fu, Zehuai Wen

**Affiliations:** ^1^Key Unit of Methodology in Clinical Research, Guangdong Provincial Hospital of Chinese Medicine, Guangzhou 510120, China; ^2^Department of Dermatology, Guangdong Provincial Hospital of Chinese Medicine, Guangzhou 510120, China; ^3^Guangdong Provincial Key Laboratory of Clinical Research on Traditional Chinese Medicine Syndrome, Guangzhou 510120, China; ^4^Department of Public Health Sciences (Global Health/IHCAR), Karolinska Institutet, 17177 Stockholm, Sweden; ^5^Department of Otorhinolaryngology, Guangdong Provincial Hospital of Chinese Medicine, Guangzhou 510120, China; ^6^Department of Acupuncture and Moxibustion, Guangdong Provincial Hospital of Chinese Medicine, Guangzhou 510120, China; ^7^Department of Nephropathy, Guangdong Provincial Hospital of Chinese Medicine, Guangzhou 510120, China; ^8^National Centre for Design Measurement and Evaluation in Clinical Research, Guangzhou University of Chinese Medicine, Guangzhou 510405, China

## Abstract

*Background*. The Sanfu herbal patch (SHP) has been widely used to treat allergic rhinitis (AR) in China. SHP has been reported to be effective for managing the symptoms of AR, but the evidence suffers from methodological limitations. Therefore, we designed a three-armed, randomized, and placebo-controlled trial to evaluate the efficacy and safety of SHP for persistent allergic rhinitis (PAR). *Methods*. The trial consists of 5 treatment sessions along with a one-year follow-up. This process is then repeated in the second and third years. Eligible participants diagnosed with PAR were randomized at a ratio of 2 : 2 : 1 into one of three groups: (a) SHP group; (b) placebo group; or (c) waiting-list group. The waiting-list group will receive no treatment in the first year but will receive SHP in the following two years. The primary outcome, total nasal symptoms score, is self-assessed at the beginning of each treatment session and during each annual follow-up. Secondary outcomes include the Rhinoconjunctivitis Quality-of-Life Questionnaire, allergic rhinitis attacks, and relief medications. The trial will be stopped if early termination criteria are met during the interim analysis. *Ethics*. This protocol has been approved by site ethics committee (number B2014-014-01) and is registered with ClinicalTrials.gov NCT02192645.

## 1. Background

Allergic rhinitis (AR) is a common condition that affects 17–29% of the population of Europe [1–3] and 9–38% of the population of China [4]. AR can be subdivided into intermittent allergic rhinitis (IAR) or persistent allergic rhinitis (PAR) as described by the Allergic Rhinitis and its Impact on Asthma (ARIA) guidelines [1, 5]. Although not life-threatening, AR causes more than the classic symptoms. It also impairs sleep, work, or school performance, as well as several other domains of quality of life [6–8].

Currently, symptomatic pharmacotherapy used to treat AR includes antihistamines, intranasal corticosteroids, leukotriene receptor antagonists, and/or decongestants [9], but these treatments tend to cause undesirable side effects [5]. If AR patients fail to respond to the typical treatments, immunotherapy is considered. However, its anaphylactic risk, prolonged duration, and relatively high cost result in poor patient compliance. Consequently, complementary and alternative medicine treatments have been sought [10], especially traditional Chinese medicine (TCM) [11–13].

TCM management of health can be characterized as holistic with the emphasis on regulating the integrity of the human bodily functions and the interaction between individuals and their environments [14, 15]. According to TCM theory, patients with AR have insufficient* Yang Qi*, a metaphysical energy with a nature of hotness inside the body [16].* Sanfu* refers to the hottest period of the year, from July to August, and is considered to be the richest time for* Yang Qi* [16]. Therefore, the* Sanfu* period is of special significance for TCM in treating AR to gain more* Yang Qi* from the environment.

The Sanfu herbal patch (SHP) is a treatment method in which processed Chinese herbal preparations are applied directly to specific acupoints during the* Sanfu* period. This is done in order to produce therapeutic effects through the combination of herbal infiltration absorption, acupoint stimulation, and time effect [16]. The herbs with a nature of warm or hot are used to cause irritation on the skin such as local redness, hotness, and/or even blisters [17]. Therefore, skin absorption and stimulation of the meridians and/or acupoints are enhanced to generate a greater effect on patients. SHP was first applied to treat AR during the* Qing* dynasty (1644–1912) [18]. Nowadays, SHP has been awarded “Intangible Cultural Heritage” status in Guangdong province, China [19], and has achieved widespread popularity in mainland China [20]. In Beijing, there were 653 medical institutions using SHP in 2014 according to official data [21]. In addition, it was reported that the number of people receiving SHP in one TCM general hospital increased at an annual rate of 10% in recent years, to about 23,000 people in 2013 [22]. Due to the noninvasive and easy-to-manipulate nature of the treatment, SHP has also been increasingly used in TCM clinics in Taiwan [17, 23].

Several studies have been conducted to explore the efficacy of SHP for AR patients. Tai's observational study on SHP for patients who were diagnosed with allergic diseases indicated that SHP was effective among 60% of AR patients after treatment [17, 23]. However, patient-reported diagnosis of AR and uncertain criteria for determining treatment efficacy limit the interpretation of the results. Preclinical and clinical studies have shown that SHP is beneficial to AR via immunological mechanisms by decreasing the serum Immunoglobulin E (Ig E) [24–27]. Furthermore, a number of randomized controlled trials (RCTs) conducted in mainland China have demonstrated a promising effect of SHP in treating AR [28–43]. Three of them have observed a significant improvement in nasal symptoms and/or quality of life among patients receiving an SHP treatment, compared to those receiving a placebo [41–43]. In addition, an RCT conducted in Taiwan evaluated SHP versus a placebo and showed that SHP is effective, especially for nasal symptoms, and caused a significant improvement in patients' quality of life [28].

A recently published systematic review confirmed the effect of SHP. However, it also pointed out that previous RCTs have suffered from a variety of methodological limitations, including absence of sample size calculation, inappropriate control groups, and lack of clarity about the types of AR [20]. The Sanfu Herbal Patch Clinical Application Guidelines in China recommend three treatment sessions (TSs) per year for three consecutive years for treatment of AR [44]. However, most RCTs apply only three to five TSs, with or without a follow-up period [20]. A deficiency is evident even in a high-quality RCT [28] in which SHP was used for only 33 AR patients over an eight-week treatment period including six TSs. Furthermore, the outcome measurements were evaluated immediately after treatments at the 8th week, which could have produced bias without follow-up in AR high risk period. The safety issues of SHP could not be better addressed due to the short duration as well. This study also showed a significant improvement in nasal obstructions after treatment in the placebo group, which indicates a placebo effect (PE) in the treatment of AR. Therefore, a large-scale and rigorously designed RCT which overcomes identified methodological problems is necessary. The primary objective of this study is to evaluate the efficacy of SHP with a focus on the nasal symptoms in patients with PAR. Secondary objectives include an evaluation of whether SHP (1) can improve patients' quality of life, (2) can reduce the frequency of the allergic rhinitis attacks (ARAs), and (3) can alter the amount of relief medications (RMs) used. If the efficacy of SHP is confirmed, the PE of the SHP is further explored.

## 2. Methods

### 2.1. Design and Setting

**Figure 1:**
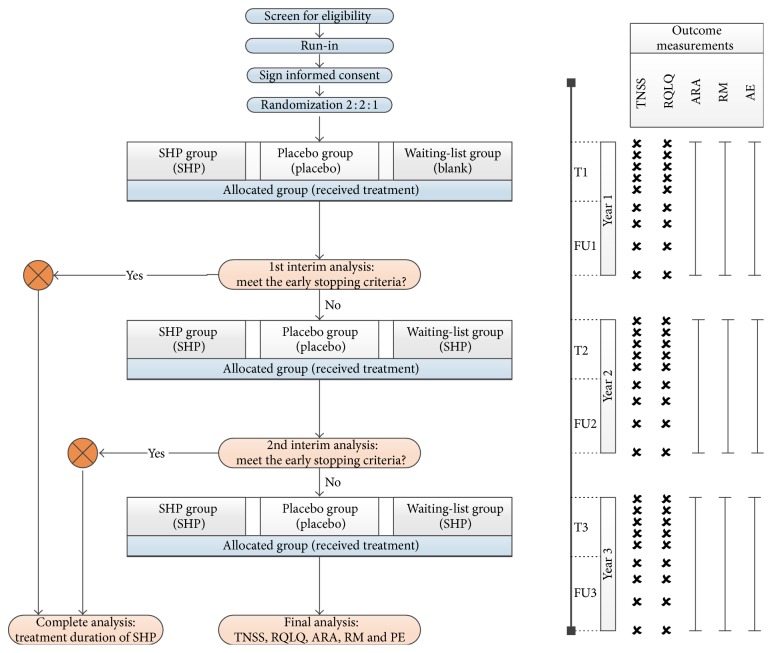
Trial flow diagram in the SPAR study. SPAR: Sanfu herbal patch at acupoints for persistent allergic rhinitis, SHP: Sanfu herbal patch, TNSS: Total Nasal Symptom Score, RQLQ: rhinitis quality-of-life questionnaire, ARA: allergic rhinitis attack, RM: relief medication, AE: adverse event, PE: placebo effect, T1: treatment in the first year, FU1: follow-up in the first year, T2: treatment in the second year, FU2: follow-up in the second year, T3: treatment in the third year, and FU3: follow-up in the third year.

This is a three-armed, randomized, and placebo-controlled trial, conducted in Guangdong Provincial Hospital of Chinese Medicine (GPHCM), Guangzhou, China. A flow chart of this trial is provided in Figure 1. The trial consists of 5 TSs conducted during the* Sanfu* period along with a one-year follow-up. This process is then repeated in the second and third years after treatment is begun. After providing written informed consent, eligible participants were randomized at a ratio of 2 : 2 : 1 into one of three groups: an SHP group receiving SHP treatment; a placebo group receiving placebo patch treatments; a waiting-list group receiving no treatment in the first year but then receiving SHP treatments the following two years. To allow for early termination, the trial will be assessed in two equally spaced interim analyses at the end of the first and second years, respectively. The trial will be stopped if a statistically significant difference between SHP and placebo is obtained during the interim analysis.

### 2.2. Participants

Participants were recruited via local advertising, local newspapers, and doctor referrals from otorhinolaryngology clinics in GPHCM. Interested individuals need to contact research assistants by phone or email. Trial information and consent forms were sent to them to read prior to scheduling their first visit. In the first visit, screening evaluations were conducted and recorded to ensure the eligibility of each individual. For each participant who was eligible and willing to participate in the trial, research assistants obtained a signed consent form.

For inclusion in this trial, participants had to meet the following criteria: aged ≥ 18 with PAR, defined clinically with symptoms being present at least 4 days a week, for at least 4 weeks [5]; having positive serum specific Ig E tests with results ≥ 0.35 IU/mL on mites (*Dermatophagoides pteronyssinus* or* Dermatophagoides farinae*), cockroach (*Blattella germanica*), or house dust; and having TNSS ≥ 3.

Main exclusion criteria for participants included one or more of the following: seasonal or chronic instance of other forms of rhinitis (i.e., sinusitis); asthma and/or moderate-to-severe atopic dermatitis; nasal structural abnormalities; diabetes mellitus requiring insulin injections; antiallergy treatment at present due to asthma, eczema, atopic dermatitis, or other diseases; serious acute or chronic organic disease or mental disorders; autoimmune disorders including the application of immunosuppressant or HIV infection; specific immunotherapy for >3 years; systemic corticosteroids treatment at time of treatment, or within the previous 6 months; intranasal corticosteroids at time of treatment, or within the previous 15 days; pregnancy or breast feeding; concurrent involvement in other clinical trials. Further exclusion criteria included blood coagulation dysfunction and/or use of antithrombotics at time of treatment and any TCM therapy such as acupuncture, moxibustion, and/or Chinese herbal medicine at time of treatment or within the previous month.

### 2.3. Randomization and Allocation Concealment

A block randomization sequence was generated by SAS 9.2 software (SAS Institute Inc., Cary, USA), which was performed by GPHCM's Key Unit of Methodology in Clinical Research (KUMCR). Eligible participants were randomly assigned to either the SHP group, the placebo group, or the waiting-list group at a ratio of 2 : 2 : 1. An independent researcher prepared treatment cards, on which a serial number and one of three names were printed, each representing one of the three groups. This person was also responsible for attaching a label including the name to the corresponding tube which was filled with either herbal or placebo ointment. Treatment allocations were stored in password-protected files and held independently by a staff of KUMCR. While receiving the first treatment, participants were given sequential treatment cards from independent researchers to ensure adequate concealment. Participants were then allocated into one of three groups according to the name printed on their treatment card. However, it was not possible to conceal the allocation among the waiting-list group.

### 2.4. Blinding

The participants, operational assistants, acupuncturists, research nurses, data managers, and statisticians do not know the treatment allocations, which will not be revealed until the end of study. However, blinding of the waiting-list group is not possible for this trial. For each participant, operational assistants prepare the patches by using ointment from tubes labeled with the same name as that on the participant's treatment card. The placebo patch is identical in appearance to the SHP. The implementation of treatments is performed by two acupuncturists using the patches prepared by the operational assistants. Due to the nature of SHP, it is difficult to ensure blinding among participants allocated to the SHP and placebo groups. Therefore, participants are required to wait for 60 minutes in a room, after which their treatment patches are torn off by research nurses. In addition, acupuncturists, operational assistants, and research nurses are instructed not to communicate with participants about the possibility of their allocation.

### 2.5. Intervention

**Figure 2:**
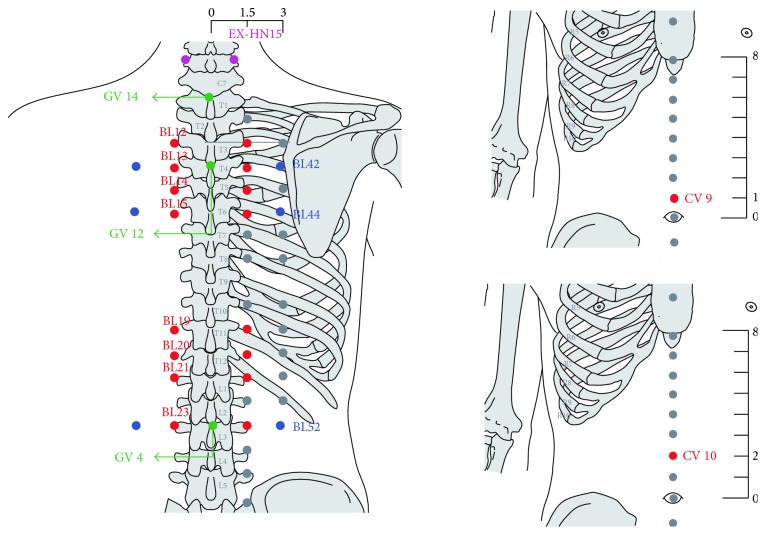
Diagram of prescribed acupoints from the WHO Standard Acupuncture Point Locations in the Western Pacific Region in the SPAR^(1)^ study in 2014. ^(1)^SPAR: Sanfu herbal patch at acupoints for persistent allergic rhinitis.

The complete formula for SHP is not publicly available, but the primary herbal ingredients include* Semen sinapis*,* Herba asari*,* Radix kansui*,* Rhizoma corydalis*, and* Ephedra sinica*. The herbs are processed into powder and mixed with fresh ginger juice to create SHP ointment which is then mechanically poured into tubes. The resulting SHP ointment is usable for a period of 24 hours after production. The placebo ointment is composed of flour, buckwheat flour, food colorants, and water, resulting in an ointment similar in appearance to the SHP ointment. The SHP and matching placebo are manufactured by the Pharmaceutical Preparation Department in GPHCM that meets the requirements of the regulatory guidance issued by the China Food and Drug Administration. Approximately two grams of ointment is squeezed by operational assistants onto a circular fabric, 5 centimeters in diameter for each acupoint. There are 5 TSs per year from 2014 to 2016, and the exact dates for each session are calculated in accordance with the Chinese Lunar Calendar (Table 1). In each TS, a group of ten acupoints are used. Acupoints are accessible only one week before the TS of a given year (Table 1, Figure 2).

Participants in the SHP group receive SHP during each TS according to the following procedure. Each participant is asked to expose their back and abdomen. One piece of SHP is attached to each acupoint by one of two acupuncturists from the Department of Acupuncture and Moxibustion in GPHCM. Participants are then asked to wait in a room for 60 minutes, and research nurses are required to carefully observe them. If a participant feels any unbearable pain or a burning sensation, the SHP is to be removed immediately by nurses. Otherwise, each TS is to last up to 60 minutes. Exact treatment duration of each participant is recorded in the treatment cards. For participants in the placebo group, placebo patches are applied during each TS, and the procedures are similar to those in the SHP group. Participants in the waiting-list group receive neither SHP nor placebo patches for the first year but will receive the same treatment as the SHP group for the following two years. If early termination criteria are met, all three groups will receive SHP for the next one or two years.

Participants from all three groups are instructed to stop symptomatic relief RMs during the one-week run-in and treatment periods. However, they are allowed to take RMs if needed during the follow-up period. These RMs are required to be documented in participants' diaries.

### 2.6. Outcome Measurement

For the evaluation of the primary and secondary outcome measures, participants are required to complete two questionnaires, the Total Nasal Symptom Score (TNSS) and the Rhinoconjunctivitis Quality-of-Life Questionnaire (RQLQ), at the beginning of each of five TSs (from 2nd to 7th weeks) and at the 8th, 19th, 33rd, and 52nd weeks during each annual follow-up. In addition, participants are asked to complete diaries throughout the trial. The frequency of the ARAs is recorded and the use of RMs or other medications is documented in detail (Figure 1).

The primary outcome is the change of the TNSS from baseline to the end of the 52nd week. TNSS evaluates four nasal symptoms: nasal obstruction, sneezing, rhinorrhea, and nasal itch. The symptoms are self-assessed and recorded by participants, using a five-point scale (0 = no symptoms; 1 = mild symptoms; 2 = moderate symptoms; 3 = severe symptoms; 4 = very severe symptoms) [45]. The TNSS ranges from 0 to 16, with low scores indicating lighter nasal symptoms.

Secondary outcomes include (1) the change of RQLQ score from baseline to the 8th, 19th, 33rd, and the 52nd weeks; (2) response to interventions, defined as participants with a change in RQLQ score of ≥0.5 from baseline; (3) the frequency of ARAs; and (4) the use of RMs. The PE of the SHP is further explored based on the TNSS and RQLQ score between the placebo group and the waiting-list group at the end of the 1st year.

### 2.7. Safety Assessment

An independent Data and Safety Monitoring Committee (DSMC) evaluates the progress of the trial and assesses the safety data which may be requested during the trial. Adverse events (AEs) are defined as any undesirable experience occurring to participants during the trial period, whether associated with the intervention or not. Participants are instructed to report any AE to the research team at any time. All details of AEs such as time of occurrence, severity, management, and causality to the intervention are recorded on case report forms (CRFs). The common AEs related to SHP include local itching, redness, and blisters [17]. The causality between AEs and intervention is assessed according to the WHO Uppsala Monitoring Centre System for Standardized Case Causality Assessment [46]. All AEs are followed up from the date they are brought to the investigator's attention until the AE has been resolved. Severe AEs or adverse drug reactions (ADRs) are defined according to the International Conference on Harmonization (ICH) guideline [47] and must be reported to both the DSMC and the Ethics Committee of GPHCM within 24 hours. In the event of an emergency medical situation, the individual's randomization code and group allocation can be identified via a standard operational procedure.

### 2.8. Quality Control

Before recruitment, the whole research team including acupuncturists, operational assistants, and research nurses were required to attend a training workshop. This was done before the trial to ensure their strict adherence to the study protocol and familiarity with the trial administration process. They are also provided with a written protocol and standard operation procedure documents. The two acupuncturists who apply the treatment each have acupuncture licenses from the Ministry of Health of the People's Republic of China and have over five years of experience in the application of SHP.

The data collected in this trial comprises information recorded in CRFs and information on the TNSS and RQLQ. Data is entered using the double-entry method. Data quality is checked regularly by research assistants and overseen by monitors. Data monitoring is conducted regularly with standard operation procedures by the Guangdong International Clinical Research Center of Chinese Medicine (Guangzhou, China). Audits are performed regularly by the GPHCM Department of Science Research. All modifications are marked on the CRFs. Data managers then recheck the data before logging it and promptly notify the research team if any discrepancy was found. The database is locked after all data has been cleaned. If participants withdraw from the trial either during the treatment period or the follow-up phase, the reasons should be clarified only if they are willing and the rate is analyzed via statistics.

### 2.9. Sample Size Calculation

We used a group sequential test to compare the SHP and placebo groups, hypothesizing a difference in the level of TNSS between SHP and the placebo. The sample size was calculated based on a mean change of 1.47 with a standard deviation (SD) of 2.9 scores in the SHP group and a mean change of 0.7 (SD: 1) scores in the placebo group [28]. We applied a 2 : 2 : 1 ratio to the SHP group, placebo group, and waitlist group, respectively. Considering a two-tailed significance level of 5% and a power of 80%, the sample size was 140 for the SHP group, 140 for the placebo group, and 70 for the waiting-list group. Assuming a dropout rate of approximately 20%, we will require a sample size of 174 for the SHP group, 174 for the placebo group, and 87 for the waiting-list group.

### 2.10. Statistical Analysis

Statistical analysis will be performed in a blinded manner by qualified statisticians using PASW Statistic 18.0 (IBM SPSS Inc., Armonk, New York, USA) and SAS 9.2 software (SAS Institute Inc., Cary, USA). Missing data will be replaced according to the principle of multiple imputations. Two-tailed *p* values < 0.05 are considered to be statistically significant. Analyses will be performed for 2 populations: (1) an intention-to-treat population with all patients randomized and treated with at least one TS and (2) a per-protocol population including only patients with no major protocol deviations.

Demographic and clinical characteristics, as well as baseline data, will be presented to assess the baseline comparability of the three groups. Descriptive statistics will be presented for each group as the mean change (SD, 95% confidence intervals) or the percentage change in the outcomes from baseline to each time point. Differences in mean change from baseline to each time point will be compared between groups using analysis of variance. Comparisons between the two groups will be conducted using the superiority test based on the Bonferroni adjustment. For the analysis of factors affecting the outcome change in each time point, mixed-effects linear or logistic regression model will be performed adjusting for baseline characteristics and other variables. Safety will be evaluated by tabulations of AEs and ADRs and will be presented with descriptive statistics for each group. A chi-square test or Fisher's exact test will be used to compare the frequency difference of AEs and ADRs among the three groups. As cases are divided into different degrees of AEs and ADRs, a rank-sum test will be performed for analyzing the independently ordered multiple category data among the three groups.

### 2.11. Interim Analysis

A group sequential design is utilized in this trial. Two equally spaced interim analyses will be performed at the end of the first and second years, respectively. Therefore, *α*-levels will be adjusted according the alpha-spending function approach in order to maintain the assumed 0.05 level for the overall study analysis. The primary purpose of the interim analysis is to determine whether the trial should be terminated early. The early terminated criterion is a statistically significant difference regarding the change of TNSS between participants receiving SHP and those receiving placebo patches at the adjusted significance level using an independent *t*-test or nonparametric tests. The first interim analysis will only analyze the difference between the SHP group and the placebo group. The second interim analysis, however, will analyze not only the difference between these two groups but also the difference between the placebo group and the waiting-list group which will have received five TSs of the SHP by the 2nd year. Independent statisticians who do not know treatment allocations will perform the interim analysis. Results of the interim analysis will be reported to the DSMC who will have unblinded access to all data. If the trial is terminated as a result of the interim analysis or by the recommendations of the DSMB, the final efficacy and safety analyses will be performed using the full dataset up until the interim end of the trial.

### 2.12. Ethical Approval

This study protocol has been approved by the Ethics Committee of GPHCM (number: B2014-014-01). It is explicitly outlined to all participants that the trial involves a waiting-list group and furthermore that informed consent to participate includes an agreement to undergo a one-year period of clinical monitoring. This is to occur prior to treatment if assigned to the waiting-list group. All participants are provided with enough time to decide whether or not to sign the informed consent. Written informed consent must be obtained from each participant before randomization.

## 3. Discussion

To our knowledge, this is the first clinical study to investigate the efficacy of SHP for PAR compared to both a placebo treatment and a waiting-list control in a 3-armed clinical trial. Compared to previous studies of SHP in the treatment of PAR, this trial has a larger participant pool, clearer diagnostic criteria of the classification of AR, a more rigorous methodology, and a longer study period.

AR can be classified as either IAR or PAR, based on the duration and severity of symptoms [5]. However, many previous trials evaluating the effect of SHP have examined AR without separating the differences between IAR and PAR [20]. In China, the majority of AR patients turn out to have persistent rhinitis with or without seasonal aggravation [48]. In addition,* D. pteronyssinus*,* D. farina*, and* B. germanica* have been found to be the most important allergens in the Chinese population [48, 49]. Taking the characteristics of AR itself into account, we classify the AR patients and only target patients with clinically diagnosed PAR in which symptoms present at least 4 days a week, for at least 4 weeks with a positive serum specific Ig E test to at least one of the three most prevalent allergens.

The total trial period will last for a period of three consecutive years, in accordance with the recommended treatment courses in the Chinese guidelines [44]. This is longer than previous studies. To allow for early termination, we will conduct two interim analyses to address possibilities including the following: (1) the SHP treatment is clearly effective [28] so that it is unnecessary to continue to the planned termination point insofar as the difference reaches statistical significance, at the adjusted significance level or (2) the SHP treatment is ineffective, but the continuation of the trial would provide a chance of reaching statistical significance [50]. In addition, the four follow-up visits per year will cover the immediate effect of SHP, as well as its long-term effects for the period with low and rapidly changing temperature. This is done because cold air and temperature change have been shown to be the two most common factors triggering nasal symptoms of AR in China [48].

Blinding offers significant advantages in the overall design of this study, as SHP is reported to have a potential PE [28]. In this trial, we blind the acupuncturists to the allocations by separating operational assistants who prepare the patches. This prevents the acupuncturists from being exposed directly to the labeled tubes filled with either SHP or placebo ointment. Although the placebo ointment is similar to the SHP ointment in appearance, it is difficult to imitate common skin irritations such as local redness, hotness, and/or blisters caused by SHP [17]. Therefore, it is a challenge to blind the participants, especially those who have experience with this treatment. However, skin reactions vary from person to person. To maintain blinding, participants will be asked to wait in a separate room. This will reduce the chances that they communicate with each other. In addition, the participating acupuncturists, assistants, nurses, and statisticians are all blinded to the allocations and instructed not to communicate with participants about the treatment reaction. Nevertheless, it must be pointed out that we cannot control the bias produced by the waiting-list group which is the open-label arm of this trial. Noncompliance from the waiting-list group may lead to a high dropout rate and/or loss of follow-up in the first year. To improve compliance, participants will be contacted by telephone, e-mail, or message before each visit point.

The therapeutic effect of SHP integrates three main elements: herbal infiltration absorption, acupoints stimulation, and time effect [16, 51]. Generally, the herbal patches for allergic diseases are produced based on the Lengxiao formula, as recorded in the* Zhang Shi Yi Tong* (*Qing* dynasty) [52]. It consists of four herbs:* Semen sinapis*,* Herba asari*,* Radix kansui*, and* Rhizoma corydalis*. Although there are many variations in the prescriptions of the medicinal herbal patches in the treatment of AR, these four herbs are reported to be the most commonly used and primary components in SHP clinical trials [20]. The herbal patches in our trial also contain these four herbs as the main components and have been further developed by Professor Wenbin Fu who is a renowned acupuncture expert in China.* Ephedra sinica* is selected as one of the primary components because it is reported to have antiallergic effect in the experimental studies by inhibiting the release of allergic mediators [53] and vascular permeability [54]. This solution has been used to treat AR at GPHCM since the 1980s and has been reported to be effective for AR [55, 56] and asthma [57].

Local irritations caused by herbal patches not only make it easier for the body to absorb the medicinal substances through the skin but also enhance the stimulation of specific acupoints. Therefore, selections of acupoints could influence the effect of SHP. A review of clinical studies of SHP showed that most of the prescribed acupoints are located in the BL meridian on the back [58]; and BL12, BL13, BL20, and BL23 are the commonly selected acupoints. In this trial, most of the acupoints adopted in 2014 are located in the BL meridian and include these four acupoints (Table 1, Figure 2). We will not use the same acupoints for each year. This may influence the standardization for this trial. However, this reflects the actual treatment applied in the real practice.

SHP requires that the treatment is applied at a special time, the* Sanfu* period, to generate a greater effect on patient. This is because the* Sanfu* period is expected to have the hottest weather of the year and is considered to be the richest in* Yang Qi* as well [52]. Xia et al. evaluated the relationship between* Sanfu* periods and the temperature-humidity index (THI) based on the daily minimum/maximum temperatures and the relative moisture observed in 432 meteorological stations in China between 1964 and 2004. The authors found that the peak THI occurred during the* Sanfu* period. The higher the THI is, the more the people feel hot and uncomfortable [59].* Sanfu* periods are calculated in accordance with the Chinese Lunar Calendar, in which days are grouped within a 10-day week. The names of the 10 days are* Jia*,* Yi*,* Bing*,* Ding*,* Wu*,* Ji*,* Geng*,* Xin*,* Ren*, and* Kui* in sequential order.* Jia* is a name like Monday in the Western 7-day week,* Yi* is like Tuesday, and* Geng* is the 7th day in the 10-day circulated system. Typically, the* Sanfu* period consists of three 10-day periods, each starting with a* Geng* day. The initial* Sanfu* begins on the 3rd* Geng* day after the summer solstice (*Xia zhi*); the middle* Sanfu* begins on the 4th* Geng* day after the summer solstice which lasts ten days but sometimes twenty; and the final* Sanfu* begins on the 1st* Geng* day after Autumn begins (*Qiu fen*) [16]. In our study, we apply treatments on the three starting* Geng* days and add two extra* Geng* days to consolidate the efficacy. One is the* Geng* day right before the initial* Sanfu*, and the other one is the* Geng* day right after the final* Sanfu* when the middle* Sanfu* is 10 days long (as what occurred in 2014) or the* Geng* between the middle and the final* Sanfu* when the middle* Sanfu* is 20 days long (as what occurred in 2015 and 2016).

This protocol describes a 3-armed randomized controlled trial to assess the efficacy and safety of SHP at acupoints in the treatment of PAR. Results of this trial will provide high-quality evidence of the therapeutic effects of SHP in alleviating nasal symptoms and improving quality of life among PAR patients.

## Figures and Tables

**Table 1:** Dates and acupoints for each treatment session from 2014 to 2016 in the SPAR^(1)^ study.

		1st session	2nd session	3rd session	4th session	5th session
2014	Dates	8 July (Extra *Geng* day)	18 July (Initial *Sanfu*)	28 July (Middle *Sanfu*)	7 August (Final *Sanfu*)	17 August (Extra *Geng* day)
	Acupoints	BL13 (bilateral) BL23 (bilateral) BL21 (bilateral) BL14 (bilateral) CV12 GV14	BL15 (bilateral) BL19 (bilateral) BL42 (bilateral) EX-HN15 (bilateral) CV10 GV4	BL12 (bilateral) BL20 (bilateral) BL44 (bilateral) BL52 (bilateral) CV9 GV12	BL13 (bilateral) BL23 (bilateral) BL21 (bilateral) BL14 (bilateral) CV12 GV14	BL15 (bilateral) BL19 (bilateral) BL42 (bilateral) EX-HN15 (bilateral) CV10 GV4

2015	Dates	3 July (Extra *Geng* day)	13 July (Initial *Sanfu*)	23 July (Middle *Sanfu*)	2 August (Extra *Geng* day)	12 August (Final *Sanfu*)
	Acupoints	Adjusted only one week before treatments based on the traditional Chinese medicine theory				

2016	Dates	7 July (Extra *Geng* day)	17 July (Initial *Sanfu*)	27 July (Middle *Sanfu*)	6 August (Extra *Geng* day)	16 August (Final *Sanfu*)
	Acupoints	Adjusted only one week before treatments based on the traditional Chinese medicine theory				

^(1)^SPAR: Sanfu herbal patch at acupoints for persistent allergic rhinitis.

## Abbreviations

AEs: Adverse events
ADRs: Adverse drug reactions
AR: Allergic rhinitis
ARAs: Allergic rhinitis attacks
CRFs: Case report forms
DSMC: Data and Safety Monitoring Committee
GPHCM: Guangdong Provincial Hospital of Chinese Medicine
IAR: Intermittent allergic rhinitis
Ig E: Immunoglobulin E
KUMCR: Key Unit of Methodology in Clinical Research
PAR: Persistent allergic rhinitis
PE: Placebo effect
RCTs: Randomized controlled trials
RMs: Relief medications
RQLQ: Rhinoconjunctivitis Quality-of-Life Questionnaire
SD: Standard deviation
SHP: Sanfu herbal patch
TCM: Traditional Chinese medicine
THI: Temperature-humidity index
TNSS: Total Nasal Symptom Score
TSs: Treatment sessions.
